# Estimation of Fetal-to-Maternal Unbound Steady-State Plasma Concentration Ratio of P-Glycoprotein and/or Breast Cancer Resistance Protein Substrate Drugs Using a Maternal-Fetal Physiologically Based Pharmacokinetic Model [Fn fn4]

**DOI:** 10.1124/dmd.121.000733

**Published:** 2022-05

**Authors:** Jinfu Peng, Mayur K. Ladumor, Jashvant D. Unadkat

**Affiliations:** Department of Pharmaceutics, School of Pharmacy, University of Washington, Seattle, Washington (J.P., M.K.L., J.D.U.) and Department of Pharmacy, The Third Xiangya Hospital, Central South University, Changsha, China (J.P.)

## Abstract

Pregnant women are frequently prescribed drugs to treat chronic diseases such as human immunodeficiency virus infection, but little is known about the benefits and risks of these drugs to the fetus that are driven by fetal drug exposure. The latter can be estimated by fetal-to-maternal unbound plasma concentration at steady state (K_p,uu,fetal_). For drugs that are substrates of placental efflux transporters [i.e., P-glycoprotein (P-gp) or breast cancer resistance protein (BCRP)], K_p,uu,fetal_ is expected to be <1. Here, we estimated the *in vivo* K_p,uu,fetal_ of selective P-gp and BCRP substrate drugs by maternal-fetal physiologically based pharmacokinetic (m-f-PBPK) modeling of umbilical vein (UV) plasma and maternal plasma (MP) concentrations obtained simultaneously at term from multiple maternal-fetal dyads. To do so, three drugs were selected: nelfinavir (P-gp substrate), efavirenz (BCRP substrate), and imatinib (P-gp/BCRP substrate). An m-f-PBPK model for each drug was developed and validated for the nonpregnant population and pregnant women using the Simcyp simulator (v20). Then, after incorporating placental passive diffusion clearance, the *in vivo* K_p,uu,fetal_ of the drug was estimated by adjusting the placental efflux clearance until the predicted UV/MP values best matched the observed data (K_p,uu,fetal_) of nelfinavir = 0.41, efavirenz = 0.39, and imatinib = 0.35. Furthermore, K_p,uu,fetal_ of nelfinavir and efavirenz at gestational weeks (GWs) 25 and 15 were predicted to be 0.34 and 0.23 (GW25) and 0.33 and 0.27 (GW15). These K_p,uu,fetal_ values can be used to adjust dosing regimens of these drugs to optimize maternal-fetal drug therapy throughout pregnancy, to assess fetal benefits and risks of these dosing regimens, and to determine if these estimated *in vivo* K_p,uu,fetal_ values can be predicted from in vitro studies.

**SIGNIFICANCE STATEMENT:**

The *in vivo* fetal-to-maternal unbound steady-state plasma concentration ratio (K_p,uu,fetal_) of nelfinavir [P-glycoprotein (P-gp) substrate], efavirenz [breast cancer resistance protein (BCRP) substrate], and imatinib (P-gp and BCRP substrate) was successfully estimated using maternal-fetal physiologically based pharmacokinetic (m-f-PBPK) modeling. These K_p,uu,fetal_ values can be used to adjust dosing regimens of these drugs to optimize maternal-fetal drug therapy throughout pregnancy, to assess fetal benefits and risks of these dosing regimens, and to determine if these estimated *in vivo* K_p,uu,fetal_ values can be predicted from in vitro studies.

## Introduction

Pregnant women frequently take drugs (medication) throughout their pregnancy to treat the mother for conditions such as hypertension or cancer or to treat the maternal-fetal pair for conditions such as human immunodeficiency virus (HIV) infection (McGowan and Shah, 2000; Mitchell et al., 2011; Haas et al., 2018). However, these drugs are often prescribed without knowledge of their fetal benefits and risks that are driven by fetal (and possibly by placental) drug exposure. Fetal drug exposure can be quantified only at delivery when simultaneous sampling of umbilical vein blood and maternal blood is possible. However, because these drug concentrations are time dependent, they need to be collected in multiple maternal-fetal dyads to allow the estimation of fetal drug exposure (Zhang et al., 2017). From these, fetal drug exposure, which is the fetal-to-maternal unbound steady-state plasma concentration ratio (K_p,uu,fetal_), can be estimated (Anoshchenko et al., 2021b). For drugs that passively cross the placenta, provided there is no fetal or placental metabolism of the drug, K_p,uu,fetal_ is easy to predict, as it will be 1.0 (Zhang et al., 2017). However, the placenta is richly endowed with efflux transporters, such as P-glycoprotein (P-gp) and breast cancer resistance protein (BCRP) at the maternal-placenta barrier, which efflux the drug from the placenta to the maternal blood. For drugs that are a substrate of these efflux transporters, K_p,uu,fetal_ will be <1, and its deviation from unity will depend on the fraction of the drug effluxed by the transporter(s) (f_efflux_). Estimation of a drug’s K_p,uu,fetal_ at term and at earlier gestational age, especially for those that are effluxed, is important for several reasons. First, it can be used to adjust dosing regimens of these drugs to optimize maternal-fetal drug therapy throughout pregnancy, provided that the f_efflux_ of the drug at each gestational age can be estimated. Such estimation is now possible given our quantification of placental transporters in the first and second trimesters as well as at term by quantitative targeted proteomics (Anoshchenko et al., 2020). Second, it can be used to assess fetal benefits and risks of these drug dosing regimens. Third, these K_p,uu,fetal_ values can be used to determine if they can be predicted from in vitro studies using the proteomics-informed efflux ratio approach, as we have done before (Anoshchenko et al., 2021b). Therefore, to fulfill the above broad goals, we estimated the *in vivo* K_p,uu,fetal_ of selective P-gp and/or BCRP substrate drugs by maternal-fetal physiologically based pharmacokinetic (m-f-PBPK) modeling of umbilical vein (UV) plasma and maternal plasma (MP) concentrations obtained simultaneously at term from multiple maternal-fetal dyads. Three drugs were studied: nelfinavir (P-gp substrate), efavirenz (BCRP substrate), and imatinib (P-gp/BCRP substrate). An m-f-PBPK model for each drug was developed and validated for the nonpregnant population and pregnant women using the Simcyp simulator (v20). Then, after incorporating placental passive diffusion clearance, the *in vivo* K_p,uu,fetal_ of the drug was estimated by adjusting the placental efflux clearance until the predicted UV/MP values best matched the observed data.

## Materials and Methods

Our search criteria for selecting the drug candidates were as follows: 1) candidate drug should be transported only by P-gp or by BCRP or by P-gp/BCRP based on extensive *in vitro* studies; and 2) *in vivo* paired UV and MP drug concentrations data should be available from a large number of maternal-fetal dyads at multiple time points over the dosing interval (or for several half-lives) after the last maternal dose. A total of three candidate drugs fulfilled these criteria: nelfinavir, which is effluxed solely by P-gp and not by BCRP (Gupta et al., 2004; Salama et al., 2005); efavirenz, which is effluxed solely by BCRP but not by P-gp (Dirson et al., 2006; Janneh et al., 2009; Peroni et al., 2011); and imatinib, which is effluxed by both BCRP and P-gp (Hamada et al., 2003; Burger et al., 2004; Oostendorp et al., 2009; Zhou et al., 2009).

### PBPK Model Simulations and Criteria for Validation

**Fig. 1.**
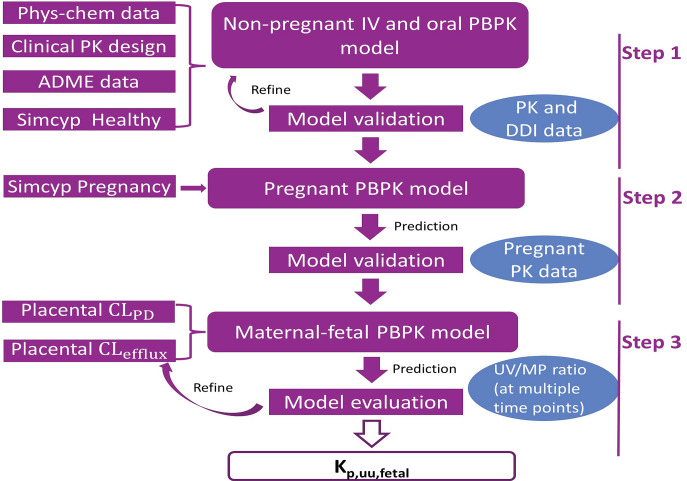
Workflow for estimation of in vivo K_p,uu,fetal_ using the Simcyp m-f-PBPK model. A PBPK model for each drug was developed for the nonpregnant population using the Simcyp simulator (v20), and the predicted PK profiles of these drugs were validated with data after intravenous (i.v.) and oral administration as well as drug-drug interaction studies (step 1). Systemic maternal PK of drugs in the second trimester, third trimester, and postpartum was predicted using the pregnant population of the Simcyp simulator and validated with the observed data (step 2). Then, using the estimated passive diffusion clearance (CL_PD_) of the drugs, the magnitude of the placental efflux clearance (CL_efflux,placenta_) and the K_p,uu,fetal_ were estimated by adjusting the CL_efflux,placenta_ until the predicted UV/MP values best matched the observed data (step 3).

PBPK simulation of the pharmacokinetic (PK) profiles of the above drugs was implemented as summarized in Fig. 1. Briefly (but detailed below), for each step of modeling, the predicted PK profiles and PK parameters (maximum plasma drug concentration [C_max_] and area under the curve of total plasma concentration-time profile [AUC]) of the drug were compared with the observed data. The observed plasma concentration-time profiles in graphical format were digitized using WebPlotDigitizer (https://apps.automeris.io/wpd/). These values were reported in the publications as geometric mean, arithmetic mean, or median. Therefore, our PBPK-predicted values are also reported in the same format. The PK profiles of the drugs were simulated using 100 virtual subjects (10 trials × 10 subjects). The PBPK model was considered validated if the observed PK profile fell within the 5th and 95th percentiles of predicted data and the simulated PK parameters fell within the range of 0.80- to 1.25-fold of the observed data (Ladumor et al., 2019a,b). All of the PBPK simulations were performed with trial designs (age range, proportion of female, gestational age, and dosing regimens) that matched the corresponding in vivo study (Supplemental Table 1).

### Development and Validation of Drug PBPK Models for Nonpregnant Adults

A full PBPK model was constructed for nelfinavir using the Simcyp simulator (v20). Drug-related parameters for nelfinavir were collected from the literature (Table 1). A whole-body PBPK model was applied for the distribution of nelfinavir, and tissue-to-plasma partition coefficient (K_p_) values were predicted using Simcyp Method 1 (Poulin and Theil, 2009). Nelfinavir binds extensively to *α*1-acid glycoprotein (AAG) with a fraction unbound in human plasma (f_u_) of 0.014 (Zhang et al., 2001; Motoya et al., 2006). Nelfinavir is metabolized by the cytochrome P450 (CYP450) isoforms CYP3A, CYP2C19, CYP2D6, CYP2C9, CYP1A2, and CYP2E1, and the fraction of drug metabolized (f_m_) by each isoform was based on the inhibition of nelfinavir metabolism in pooled human liver microsomes (HLMs) in the presence of selective cytochrome P450 inhibitors (https://www.accessdata.fda.gov/drugsatfda_docs/nda/97/020778ap.pdf). The intrinsic hepatic clearance (CL_int_) of nelfinavir by each isoform was back-calculated from the intravenous total systemic clearance (CL_iv_ = 37.7 l/h) using the Simcyp simulator (Sarapa et al., 2005) after correcting for renal clearance (f_e_ = 2%) and biliary clearance (f_CL,bile_ = 10%) (https://www.accessdata.fda.gov/drugsatfda_docs/nda/97/020778ap.pdf). Our previously reported mechanism-based inhibition and induction of CYP3A by nelfinavir in HLMs and hepatocytes, respectively (Dixit et al., 2007; Kirby et al., 2011), and competitive inhibition of CYP3A, CYP2C9, and CYP1A2 by nelfinavir (Lillibridge et al., 1998) were incorporated into the PBPK model. Then, PK data after intravenous administration were simulated and validated using the observed data. Thereafter, the Advanced Dissolution, Absorption and Metabolism (ADAM) model of Simcyp, with integrated in vitro dissolution profiles in the fed and fasted state, was used to describe nelfinavir absorption (Shono et al., 2011; Chapa et al., 2020). Then, nelfinavir PK after single oral administration in the fed/fasted state, multiple doses, and coadministration with ritonavir (inhibitor of CYP3A and CYP2D6, inducer of CYP3A and CYP2C9; Simcyp default compound file) were predicted and validated. Efavirenz and imatinib PBPK models for the nonpregnant adults were reproduced without modification from previous publications (Atoyebi et al., 2019; Adiwidjaja et al., 2020) and validated with the additional published *in vivo* data.

**TABLE 1:** Nelfinavir drug-related parameters

**Parameter**	**Unit**	**Value**	**Reference**
**Physicochemical and blood-binding properties**			
Molecular weight	g/mol	567.80	ChEMBL DrugBank
Log P_o:w_		4.07	Longer et al., 1995
Ionization pattern		Diprotic base	
pKa		6,11.06	
B/P		1.00	Zhang et al., 2001
F_u_		0.014	
Plasma binding component		AAG	Motoya et al., 2006
**Absorption phase**			
Model		ADAM	
P_app_	10^−6^ cm/s, Caco2	7.11	Kim et al., 1998
Solubility	mg/ml	4.50	Longer et al., 1995
**Distribution phase**			
Prediction method		Full PBPK model Method 1	
Vss	l/kg	2.00 for healthy5.20 for pregnancy	Predicted by Simcyp
**Elimination phase**			
CL_iv_	l/h	37.70	Sarapa et al., 2005
CL_int,CYP3A_ (f_m,CYP450_)	*μ*l/min/pmol CYP450	1.30 (25.19%)	
CL_int,CYP2C19_ (f_m,CYP450_)	*μ*l/min/pmol CYP450	29.62 (15.99%)	
CL_int,CYP2C9_ (f_m,CYP450_)	*μ*l/min/pmol CYP450	0.90 (8.72%)	
CL_int,CYP1A2_ (f_m,CYP450_)	*μ*l/min/pmol CYP450	0.99 (6.30%)	
CL_int,CYP2E1_ (f_m,CYP450_)	*μ*l/min/pmol CYP450	1.43 (11.63%)	
CL_int,CYP2D6_ (f_m,CYP450_)	*μ*l/min/pmol CYP450	8.19 (10.17%)	
Additional HLM CL_int_ (f_m_)	*μ*l/min/mg protein	145.24 (12.00%)	
CL_int,bile_ (f_CLbile_)	*μ*l/min/million cells	26.35 (10.00%)	
CL_R_ (f_e_)	l/h	0.57 (2.00%)	
**Drug interactions**			
Inhibition			
K_inact,CYP3A_	min^−1^	0.16	Kirby et al., 2011
K_app,CYP3A_	*μ*mol/l	1.82	
K_i,CYP3A_	*μ*mol/l	4.80	Lillibridge et al., 1998
K_i,CYP2C19_	*μ*mol/l	126.00	
K_i,CYP1C19_	*μ*mol/l	192.00	
Induction			
E_max,CYP3A_		11.20	Kirby et al., 2011
EC50_CYP3A_	*μ*mol/l	6.50	

ADAM, Advanced Dissolution, Absorption, and Metabolism model; B/P, blood-to-plasma partition ratio; CL_int,bile_, intrinsic biliary clearance; CL_int,CYPx_, intrinsic clearance via the listed CYP450 isozyme; CL_iv_, intravenous clearance; CL_R_, renal clearance; EC50_CYP3A_, nelfinavir concentration that produces half-maximal induction of CYP3A; E_max,CYP3A_, maximal fold induction of CYP3A relative to control; f_CLbile_, fraction of drug excreted in the bile; f_e_, fraction of drug excreted in the urine; f_m,CYP450_, fraction metabolized by CYP450 enzymes; f_u_, unbound fractions in plasma; K_app,CYP3A_, concentration of mechanism-based inhibitor associated with half-maximal inactivation rate of CYP3A enzymes; k_i,CYPx_, concentration of inhibitor that produces half-maximal inhibition of CYP450 isozyme; k_inact,CYP3A_, maximum inactivation rate of CYP3A; pKa, acid dissociation constant; P_o:w_, octanol-water partition coefficient; Vss, steady-state volume of distribution.

### Development and Validation of Drug PBPK Models for Pregnant Women

After validating the PK of the drug in the nonpregnant population, drug-specific parameters were fixed, and except for the changes in CYP450 activity, the pregnancy-induced changes in physiologic parameters specified in the Simcyp pregnancy module were implemented. The pregnancy-induced changes in hepatic CYP450 activity were based on our previously published data: CYP3A was induced 2-fold during the second and third trimesters (Ke et al., 2012; Zhang et al., 2015), CYP2D6 was induced 1.9- and 2-fold during the second and third trimesters, CYP1A2 was suppressed by 48% and 65% during the second and third trimesters (Ke et al., 2013), CYP2B6 activity was induced by 1.1- and 1.3-fold during the second and third trimesters, and CYP2C9 activity was induced by 1.5- and 1.6-fold during the second and third trimesters (Ke et al., 2014). CYP2C19 activity was suppressed by 62% and 68% during the second and third trimesters (Dickmann and Isoherranen, 2013; Ke et al., 2014). Then, nelfinavir and efavirenz PK in postpartum, second, and third trimester women was predicted and validated using the observed data. Corresponding in vivo data for imatinib are not available. We assumed physiologic parameters in postpartum women (6–12 weeks) had returned to levels in the nonpregnant women prior to pregnancy (gestational age = 0). In addition, the gestational stage in our study was defined per U.S. Department of Health and Human Services (HHS) recommendations: 1–12 weeks for the first trimester, 13–28 weeks for the second trimester, and 29–40 weeks for the third trimester.

### Estimating Human K_p,uu,fetal_ at Term

Maternal pharmacokinetics of nelfinavir, efavirenz, and imatinib (by mouth) were predicted using pregnancy PBPK models and compared with the observed PK profiles. Then, the bidirectional placental passive diffusion clearance (CL_PD,placenta_) of the drug at maternal-placental and placental-fetal barriers was estimated, as we have previously described (Zhang and Unadkat, 2017). Briefly, we chose midazolam as an in vivo calibrator to estimate CL_PD,placenta_ of nelfinavir, efavirenz, or imatinib. The CL_PD,placenta_ of the drug (nelfinavir, efavirenz, or imatinib) was estimated by scaling CL_PD,placenta_ of midazolam (CL_PD,midazolam_) using eq. 1:

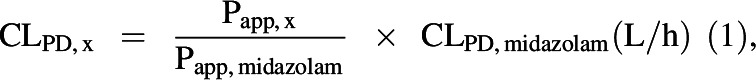



Where P_app,midazolam_ and CL_PD,midazolam_ are 489.9 nm/s and 500 l/h (mean value in Caco-2 and MDR1-MDCKI cells), respectively (Yamashita et al., 2000; Mahar Doan et al., 2002; Tolle-Sander et al., 2003; Gertz et al., 2010), and P_app,x_ is the apparent membrane permeability (P_app_) values (nm/s) of nelfinavir (8.8 in LLC-PK cells; Kim et al., 1998), efavirenz (45.85, mean value of two studies in Caco-2 cells; Takano et al., 2006; Siccardi et al., 2012), and imatinib (6.36 in MDCK II mock cells; Breedveld et al., 2005). Bidirectional intrinsic placental passive diffusion clearance (CL_int,PD,placenta_, *μ*l/min/ml placenta volume) at maternal-placenta and placenta-fetal barriers was obtained by dividing CL_PD,placenta_ by placental volume. The placental volume was calculated using eq. 2 (Kapraun et al., 2019),

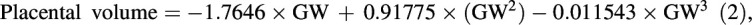

where GW is the gestational age (in weeks). After incorporating CL_int,PD,placenta_, we predicted the umbilical vein plasma concentrations and estimated the drug K_p,uu,fetal_ (eq. 3) by adjusting the intrinsic placental efflux clearance of the drug at the maternal-placenta barrier (CL_int,P-gp,placenta_ for nelfinavir, CL_int,BCRP,placenta_ for efavirenz, and CL_int,efflux,placenta_ for imatinib) until the predicted UV/MP values best matched the observed data (AAFE = 1.0) using the permeability-limited placenta model of Simcyp. The absolute average fold error (AAFE) in the predictions of UV/MP values was calculated as per eq. 4:

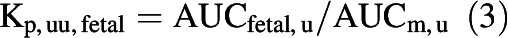


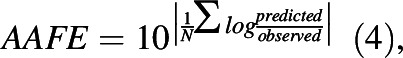

where AUC_fetal,u_ is the area under the curve of the unbound umbilical vein plasma concentration-time profile, AUC_m,u_ is the area under the curve of the unbound maternal plasma concentration-time profile, and N is the number of observed and predicted UV/MP values.

### PBPK Model Prediction of K_p,uu,fetal_ of the Drugs at an Earlier Gestational Ages (GW15 and GW25)

To predict the K_p,uu,fetal_ of nelfinavir and efavirenz at an earlier gestational age, total placental P-gp and BCRP abundance, previously quantified by us using quantitative targeted proteomics (Anoshchenko et al., 2020), was incorporated into the Simcyp pregnancy module “Sim-Pregnancy.” A second-order polynomial model was fitted to the gestational age-dependent relative abundance of placental P-gp and BCRP (relative to term value, which was set as 1.0), respectively (see eq. 5 and 6; R-square values of the fitted polynomials were 1.0; Supplemental Fig. 1).

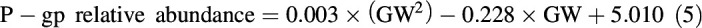


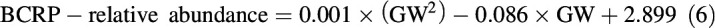



These equations were used to interpolate the placental abundance of the transporters at GW15 and GW25. Then, these interpolated values were used to scale the above estimated (term) placental efflux clearances of nelfinavir and efavirenz (CL_int,P-gp,placenta_: nelfinavir; CL_int,BCRP,placenta_: efavirenz) and incorporated in the Simcyp pregnancy module. Within this module, the above-estimated term CL_int,PD,placenta_ and CL_int,efflux,placenta_ was scaled based on the mean volume of the placenta for the respective gestational age. Then, the maternal-fetal PK profiles of the drugs were predicted at GW15 and GW25 using the same trial design as for term. From these profiles, the K_p,uu,fetal_ of nelfinavir and efavirenz was estimated. Such predictions for imatinib were not possible, as the fraction of imatinib transported by P-gp or BCRP is unknown and will need to be determined, as we have described previously (Kumar et al., 2021).

## Results

### PBPK Model Predictions and Validation for the Nonpregnant Population

**Fig. 2.**
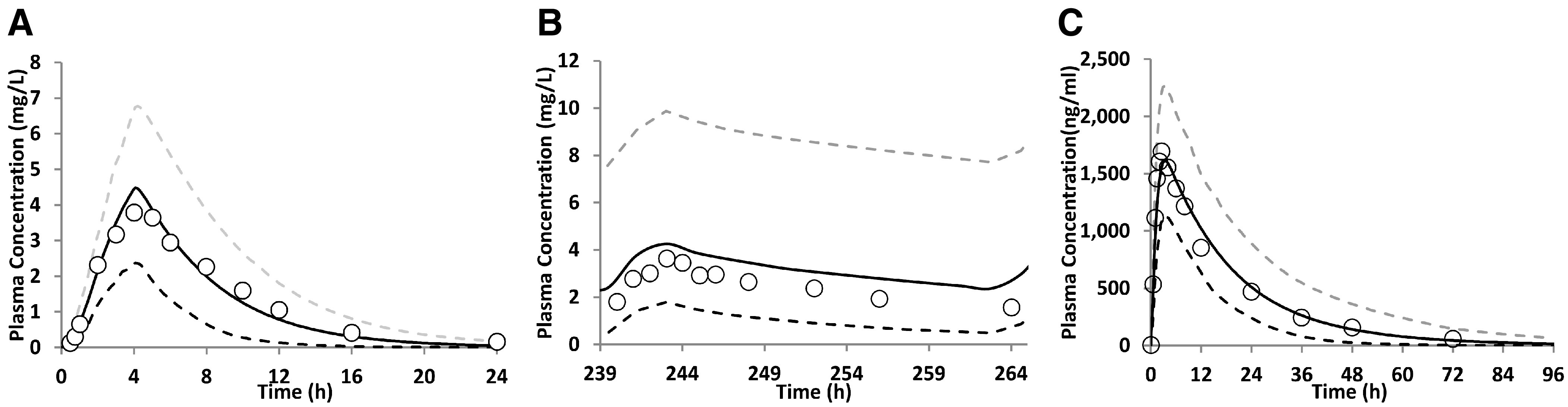
Predicted and observed plasma concentration-time (C-T) profiles of nelfinavir, efavirenz, and imatinib in the nonpregnant adults. (A) Observed (geometric mean) and predicted plasma C-T profile after single oral dose of nelfinavir (1250 mg) in nonpregnant adults (Sarapa et al., 2005; Damle et al., 2006); (B) Observed (mean) and predicted plasma C-T profile of 600 mg efavirenz (once daily by mouth) at steady state in nonpregnant adults (Villani et al., 1999); and (C) Observed (median) and predicted plasma C-T profile after single dose of 100 mg imatinib in nonpregnant adults (Ostrowicz et al., 2014). The observed data (open circles) fell within the 5th and 95th percentiles (dashed lines) of the predicted data (continuous black line). The predicted PK endpoints (AUC and C_max_) also fell within 0.80- to 1.25-fold of the observed data (Tables 2 and 3).

Our predictions of nelfinavir PK were successfully validated after intravenous dose, single oral dose (fed and fasted), multiple oral dose administration, and coadministration with ritonavir. The observed concentration-time (C-T) profiles fell within the 5th and 95th percentiles of predicted data (Fig. 2A; Supplemental Fig. 2), and the predicted PK parameters (AUC and C_max_) also fell within 0.80- to 1.25-fold of the observed data (Table 2). The PBPK models for efavirenz and imatinib were successfully reproduced, and except for imatinib C_max_ after coadministration with ketoconazole, their simulated PK profiles were consistent with the reported in vivo data (Fig. 2, B and C; Supplemental Fig. 3; Table 3).

**TABLE 2:** Observed and PBPK model-predicted plasma pharmacokinetics of nelfinavir in nonpregnant adults One hundred virtual subjects (10 trials × 10 subjects) were simulated for each study.

	I.V. Infusion (Day 1)*^a^*			I.V. Infusion (Day 11)*^b^*			Single Oral 1250 mg (Day 1)			Oral 1250 mg 2× Daily (Day 15)			Reference
Parameters	Observed	Predicted	**Ratio**	Observed	Predicted	**Ratio**	Observed	Predicted	**Ratio**	Observed	Predicted	**Ratio**	
**N**	**6**			**6**			**6**			**12**			Sarapa et al., 2005; Damle et al., 2006
AUC_last_ (mg•h/l)	23.60	26.74	**1.13**	29.20	31.88	**1.09**	26.20	26.94	**1.03**	33.70	35.06	**1.04**	
C_max_ (mg/l)	24.30	19.33	**0.80**	24.40	20.19	**0.83**	4.18	4.25	**1.02**	5.13	5.55	**1.08**	
	Single Oral 1250 mg (Fed)			Single Oral 1250 mg (Fasted)			1250mg Nelfinavir + 100 mg Ritonavir Oral 2× Daily (14 Days)						Reference
Parameters	Observed	Predicted	**Ratio**	Observed	Predicted	**Ratio**	Observed	Predicted	**Ratio**					
**N**	**52**			**52**			**12**						Kurowski et al., 2002; Kaeser et al., 2005
AUC_last_ (mg•h/l)	32.90	26.41	**0.80**	4.84	4.91	**1.01**	31.80	28.91	**0.91**				
C_max_ (mg/l)	4.30	4.36	**1.01**	0.81	0.87	**1.07**	4.09	4.67	**1.14**				

AUC_last_, AUC from time 0 to time of last measurable concentration; N, number of subjects of observed data; Ratio, Predicted/Observed values of AUC_last_ or C_max_.

*^a^*Single 30-min i.v. infusion of 1 mg nelfinavir.

*^b^*11 days oral 1250-mg dose of nelfinavir with food followed by single 30-min i.v. infusion of 1 mg nelfinavir.

**TABLE 3:** PK profiles of efavirenz and imatinib in nonpregnant population One hundred virtual subjects (10 trials × 10 subjects) were simulated for each study.

		400 mg Once Daily			600 mg Once Daily			600 mg Once Daily			Reference
	Parameters	Observed	Predicted	**Ratio**	Observed	Predicted	**Ratio**	Observed	Predicted	**Ratio**	
Efavirenz	**N**	**311**			**295**				**11**		Villani et al., 1999; Dickinson et al., 2016
	AUC_last_ (mg.h/l)	49.20	51.81	**1.05**	67.20	70.97	**1.06**	57.15	70.84	**1.24**	
	C_max_ (mg/l)	2.52	3.00	**1.19**	3.66	4.20	**1.15**	4.00	4.21	**1.05**	
Imatinib		60-Min I.V. Infusion (100 mg)			Capsule (400 mg)			Oral Solution (400 mg)			Reference
	Parameters	Observed	Predicted	**Ratio**	Observed	Predicted	**Ratio**	Observed	Predicted	**Ratio**		
	**N**	**4**			**4**			**4**			Peng et al., 2004
	AUC_inf_ (ng•h/ml)	7836.00	8098.00	**1.03**	32640.00	30971.88	**0.95**	30729.00	30971.88	**1.01**	
	C_max_ (ng/ml)	1206.00	1689.60	**1.40**	1822.00	1560.67	**0.86**	1848.00	1539.54	**0.83**	
		Oral (100 mg)			Oral (400 mg)			Imatinib (200mg) + Ketoconazole (400mg)			Reference
	Parameters	Observed	Predicted	**Ratio**	Observed	Predicted	**Ratio**	Observed	Predicted	**Ratio**	
	**N**	**37**			**37**			**14**			Dutreix et al., 2004; Ostrowicz et al., 2014
	AUC_inf_ (ng•h/ml)	6104.00	6449.95	**1.06**	24304.00	27031.78	**1.11**	19667.00	19801.34	**1.01**	
	C_max_ (ng/ml)	370.00	354.69	**0.96**	1439.00	1446.27	**1.01**	1213.00	866.52	**0.71**	

AUC_inf_, AUC from time 0 extrapolated to infinity; AUC_last_, AUC from time 0 to time of last measurable concentration; N, number of subjects of observed data; Ratio, Predicted/Observed values of AUC_last_, AUC_inf_, or C_max_.

### PBPK Model Predictions and Validation for Pregnant Women

**Fig. 3.**
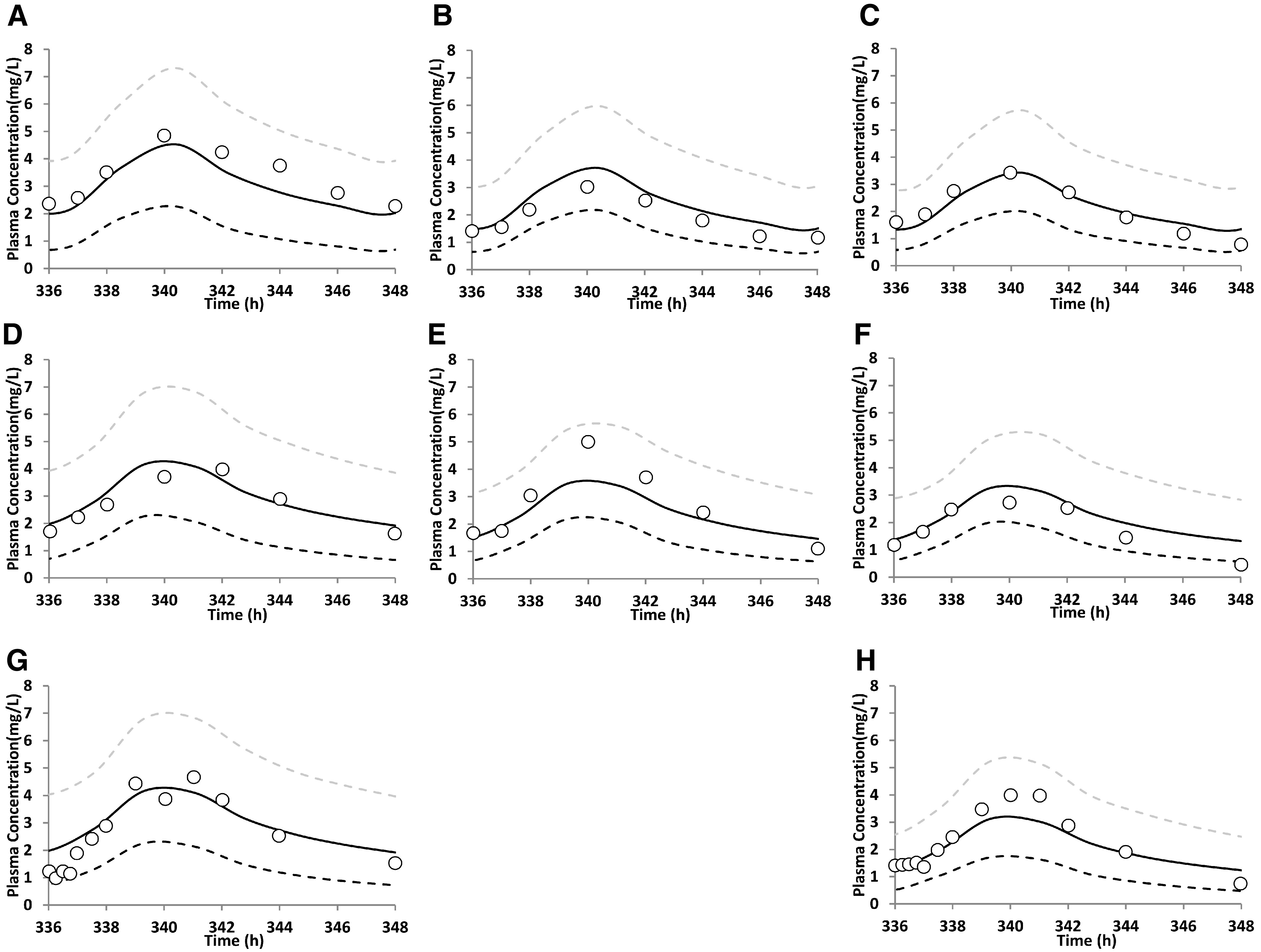
Predicted and observed plasma concentration-time (C-T) profiles of nelfinavir in pregnant women throughout pregnancy for several studies. Observed (geometric) (Fang et al., 2012) and predicted steady-state plasma C-T profile of nelfinavir (1250 mg, twice daily by mouth) in (A) postpartum, (B) second trimester, and (C) third trimester women; observed (median) (Read et al., 2008) and predicted steady-state plasma C-T profile of nelfinavir (1250 mg, twice daily by mouth) in (D) postpartum, (E) second trimester, and (F) third trimester women; and observed (geometric mean) (Van Heeswijk et al., 2004) and predicted steady-state plasma C-T profile of nelfinavir (1250 mg, twice daily by mouth) in (G) postpartum and (H) third trimester women (second trimester data are not available). The observed data (open circles) fell within the 5th and 95th percentiles (dashed lines) of the predicted data (continuous black line). The predicted PK endpoints (AUC and C_max_) also fell within 0.80- to 1.25-fold of the observed data (Table 4).

**Fig. 4.**
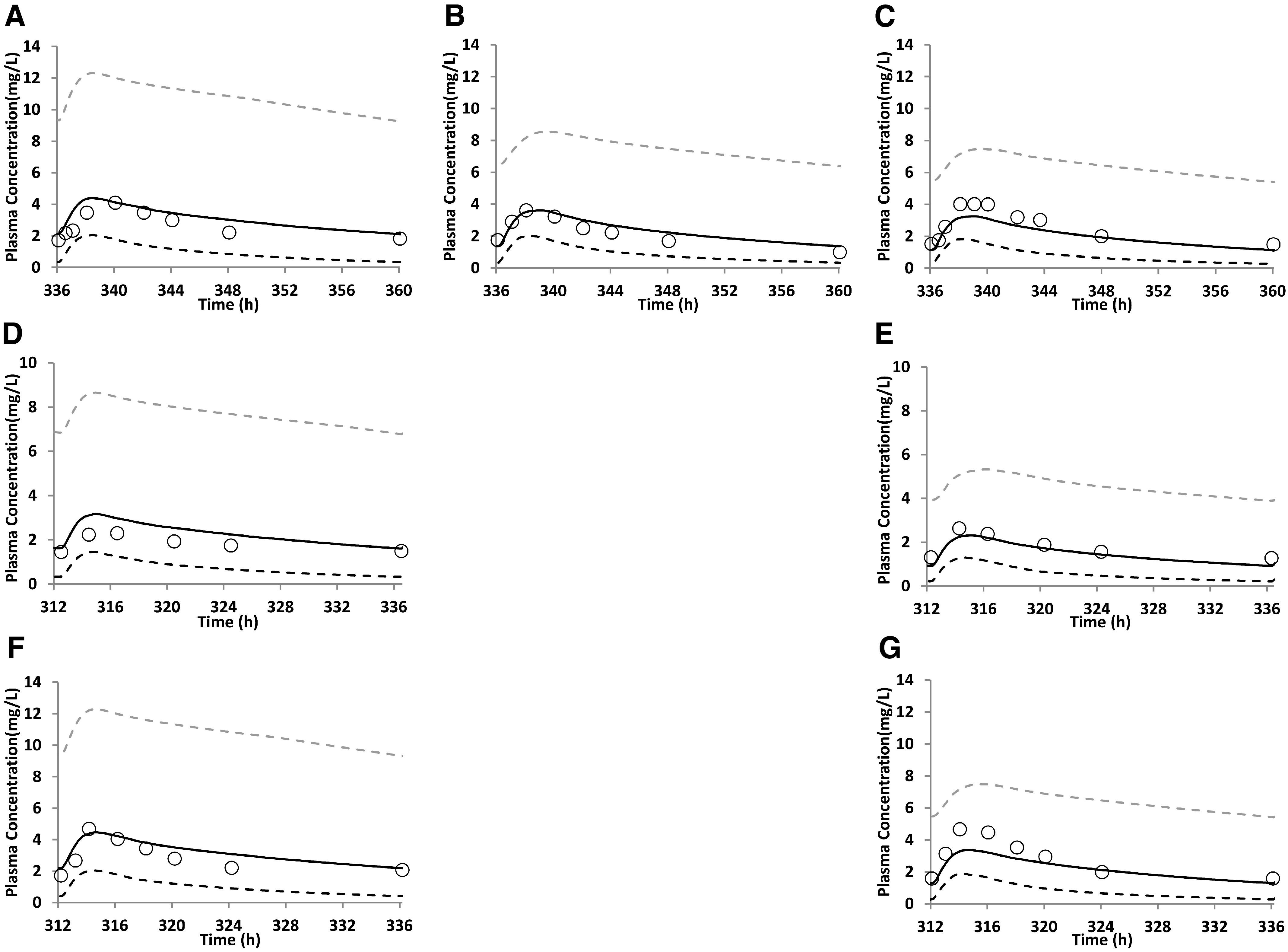
Predicted and observed plasma concentration-time (C-T) profile of efavirenz in pregnant women throughout pregnancy for several studies. Observed (median) (Kreitchmann et al., 2019) and predicted plasma C-T profile of efavirenz (600 mg, once daily by mouth) at steady state in (A) postpartum, (B) second trimester, and (C) third trimester, respectively; observed (geometric mean) (Lamorde et al., 2018) and predicted plasma C-T profile of efavirenz (400mg, once daily by mouth) at steady state in (D) postpartum and (E) third trimester (second trimester data are not available), respectively; and observed (median) (Cressey et al., 2012) and predicted plasma C-T profile of efavirenz (600 mg, once daily by mouth) in (F) postpartum and (G) third trimester (second trimester data are not available), respectively. The observed data (open circles) fell within the 5^th^ and 95^th^ percentiles (dashed lines) of the predicted data (continuous black line). The predicted PK endpoints (AUC and C_max_) also fell within 0.80- to 1.25-fold of the observed data (Table 4).

The PBPK pregnancy model for nelfinavir and efavirenz successfully predicted the PK of the drugs in postpartum, second trimester, and third trimester women (corresponding data for imatinib are not available) (Figs. 3 and 4). Also, the majority of the predicted PK endpoints (AUC and C_max_) fell within 0.80- to 1.25-fold of the observed data (Table 4).

**TABLE 4:** Predicted and observed pharmacokinetics of nelfinavir and efavirenz in pregnant women One hundred virtual subjects (10 trials × 10 subjects) were simulated for each study.

	Parameters	Observed Postpartum	Predicted Postpartum	**Ratio**	Observed Second Trimester	Predicted Second Trimester	**Ratio**	Observed Third Trimester	Predicted Third Trimester	**Ratio**	Reference
Nelfinavir*^a^*	**N**	**10**			**16**			**14**			Fang et al., 2012
	AUC_last_ (mg.h/l)	38.50	33.84	**0.88**	21.60	26.98	**1.25**	20.70	24.50	**1.18**	
	C_max_ (mg/l)	5.02	4.32	**0.86**	3.32	3.58	**1.08**	3.18	3.30	**1.04**	
	**N**	**22**			**4**			**27**			Read et al., 2008
	AUC_last_ (mg.h/l)	30.80	32.52	**1.06**	27.30	27.77	**1.02**	18.90	25.06	**1.33**	
	C_max_ (mg/l)	4.60	4.24	**0.92**	4.70	3.62	**0.77**	3.20	3.37	**1.05**	
	**N**	**11**						**11**			Van Heeswijk et al., 2004
	AUC_last_ (mg.h/l)	33.50	33.46	**1.00**				25.20	23.67	**0.94**	
	C_max_ (mg/l)	5.80	4.28	**0.74**				4.51	3.21	**0.71**	
Efavirenz*^b^*	**N**	**40**			**15**			**42**			Kreitchmann et al., 2019
	AUC_last_ (mg.h/l)	62.70	73.87	**1.18**	47.3	55.13	**1.17**	60.02	48.18	**0.80**	
	C_max_ (mg/l)	4.41	4.41	**1.00**	3.87	3.61	**0.93**	5.13	3.26	**0.64**	
	**N**	**26**						**26**			Lamorde et al., 2018
	AUC_last_ (mg.h/l)	44.11	54.14	**1.23**				39.94	36.33	**0.91**	
	C_max_ (mg/l)	2.77	3.18	**1.15**				2.75	2.40	**0.87**	
	**N**	**25**						**26**			Cressey et al., 2012
	AUC_last_ (mg.h/l)	58.30	74.63	**1.28**				55.40	52.18	**0.94**	
	C_max_ (mg/l)	5.10	4.48	**0.88**				5.44	3.38	**0.62**	

AUC_last_, AUC from time 0 to time of last measurable concentration; N, number of subjects of observed data; Ratio, Predicted/Observed values of AUC_last_ or C_max_.

*^a^*Nelfinavir dosing regimen: 1250 mg twice daily with food for at least 2 weeks.

*^b^*Efavirenz dosing regimen: 400/600 mg once daily for at least 2 weeks.

### Estimated Human K_p,uu,fetal_ at Term

**Fig. 5.**
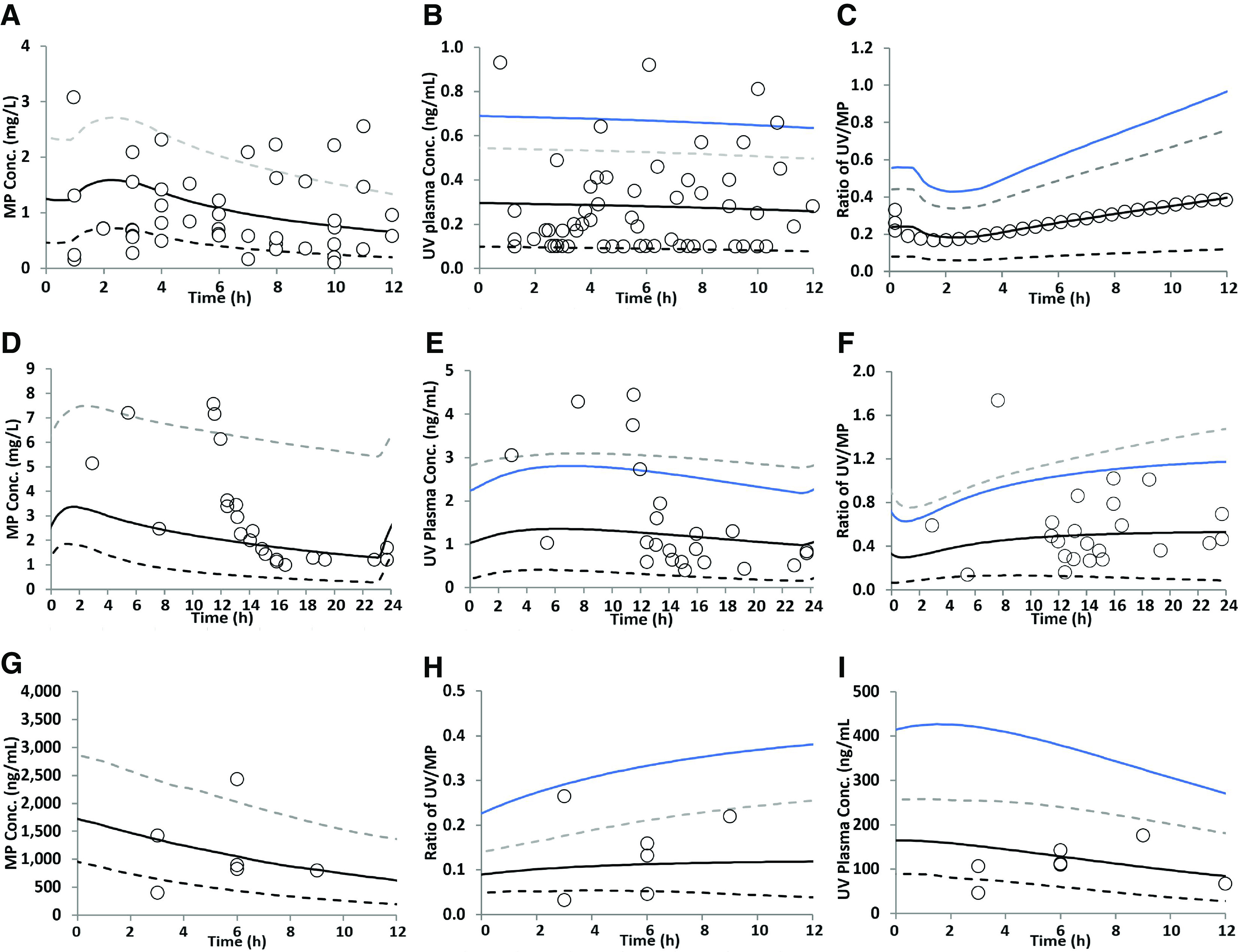
Predicted and observed (pooled) steady-state (A, D, and G) maternal plasma (MP) concentration-time profiles; (B, E, and H) umbilical vein (UV) plasma concentration-time profiles; and (C, F, and I) UV/MP profiles of the drugs with (black line) or without (blue line) in vivo placental efflux clearance. (A–C) Nelfinavir (1250 mg, twice daily) was administered (by mouth, fed) for at least 15 days, followed by 1250 mg (by mouth, fasted) on the day of delivery, between 31 and 41 weeks of gestation (Hirt et al., 2007); (D–F) efavirenz (600 mg, once daily) was administered between 37 and 41 weeks of gestation (Cressey et al., 2012); and (G–I) imatinib (400 mg daily) was administered between 35 and 41 weeks of gestation (Chelysheva et al., 2018). The x-axis is the time between the last dose and delivery. Dashed lines represent the 5th and 95th percentiles of the predicted data in the presence of CL_efflux,placenta_; open circles represent observed data. K_p,uu,fetal_ values for nelfinavir, efavirenz, and imatinib estimated from the UV/MP data were 0.41, 0.39, and 0.35, respectively.

Using our acceptance criteria, the predicted MP concentration-time profiles agreed well with the observed data of nelfinavir, efavirenz, and imatinib (Fig. 5, A, D, and G). The estimated CL_int,PD,placenta_ of nelfinavir, efavirenz, and imatinib at term were 240, 1480, and 170 *μ*l/min/ml placenta volume, respectively (Table 5). Without incorporating placental efflux clearance (CL_efflux,placenta_) that is in the presence of only CL_PD,placenta_ of the drug, the UV plasma concentration (Fig. 5, B, E, and H) and UV/MP ratio (Fig. 5, C, F, and I) were considerably overpredicted with AAFE > 1 and, as expected, the estimated K_p,uu,fetal_ was 1.0 (Table 5).

**TABLE 5:** Estimated and predicted K_p,uu,fetal_ with and without CL_efflux,placenta_

	CL_int,PD,placenta_ (*μ*l/min/ml Placenta Volume)	CL_int,efflux,placenta_ (*μ*l/min/ml Placenta Volume)	AAFE	Predicted AUC_fetal_/AUC_m_	Average Observed UV/MP Ratio (Range)	K_p,uu,fetal_			Reference
						At Term	GW25	GW15	
Nelfinavir	240	0.00	2.39	0.61	0.25 (0.05–5.18)	1.00	1.00	1.00	Hirt et al., 2007
		350.00	1.00	0.25		0.41	0.34	0.23	
Efavirenz	1480	0.00	2.21	0.95	0.49 (0.37–0.74)	1.00	1.00	1.00	Cressey et al., 2012
			2200.00	1.00	0.43		0.39	0.33	0.27
Imatinib	170	0.00	2.91	0.27	0.11 (0.05–0.22)	1.00	NA	NA	Chelysheva et al., 2018
				320.00		1.00	0.09		0.35

NA, data not available.

By adjusting CL_int,efflux,placenta_ of the drugs (nelfinavir: 350; efavirenz: 2200; imatinib: 320 *μ*l/min/ml placenta volume), the majority of the observed UV plasma concentrations and the UV/MP ratios fell within the 5th and 95th percentiles of the model predicted data (Fig. 5). As these data are steady-state data, the predicted AUC_fetal_/AUC_m_ were close to the mean observed UV/MP ratio and AAFE equaled 1.00. K_p,uu,fetal_ values at term estimated from the UV/MP data were 0.41, 0.39, and 0.35 for nelfinavir, efavirenz, and imatinib, respectively. These data indicate that the fraction of drug transported by placental P-gp or BCRP at term (f_efflux_ = 1 − K_p,uu,fetal_) followed the order imatinib (0.65) > efavirenz (0.61) > nelfinavir (0.59).

### Prediction of Nelfinavir and Efavirenz K_p,uu,fetal_ at Earlier Gestational Ages (GW15 and GW25)

**Fig. 6.**
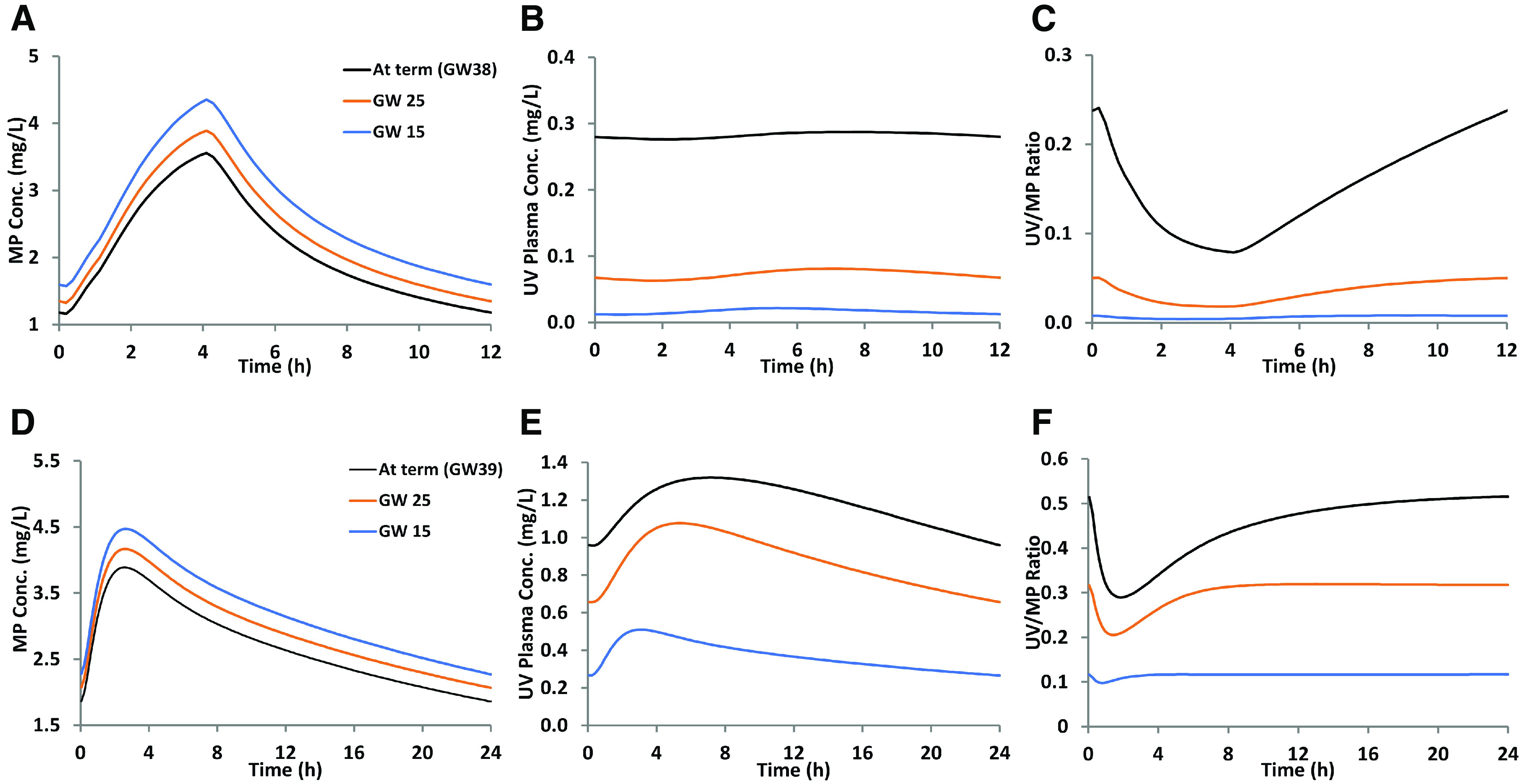
Simulated steady-state (A and D) maternal plasma (MP) concentrations; (B and E) umbilical vein (UV) plasma concentrations; and (C and F) the UV/MP profiles of (A–C) nelfinavir or (D–F) efavirenz at varying gestational ages. Profiles were simulated after administration of (A–C) nelfinavir (1250 mg, twice daily in fed state for 15 days) and (D–F) efavirenz (600 mg, once daily for 15 days). K_p,uu,fetal_ values for nelfinavir were 0.41, 0.34, and 0.23 at GWs 38, 25, and 15, respectively. K_p,uu,fetal_ values for efavirenz were 0.39, 0.33, and 0.27 at GWs 39, 25, and 15, respectively.

The MP plasma concentrations of nelfinavir and efavirenz were marginally affected by gestational age, and the UV plasma concentration, UV/MP ratio, and K_p,uu,fetal_ all decreased with gestational age (Fig. 6; Table 5).

## Discussion

Nelfinavir and efavirenz are prescribed to prevent the transmission of HIV from the mother to her fetus (Perry et al., 2005; Vrouenraets et al., 2007). However, as we have shown here, they are prevented from distribution into the fetal compartment by extensive placental efflux, thus potentially reducing their efficacy in preventing maternal-fetal HIV transmission. In contrast, imatinib, a selective tyrosine kinase inhibitor, is used to treat cancers (Ali et al., 2009). When administered to a pregnant woman, fetal harm or abortion can occur (Ali et al., 2009). These cases illustrate the importance of estimating fetal drug exposure (K_p,uu,fetal_) at all gestational ages to assess the safety and efficacy of drugs administered to pregnant women. In addition, if these safety and efficacy data dictate, these K_p,uu,fetal_ values can be used to design alternative dosing regimens to enhance drug safety and efficacy, as we have proposed for antenatal corticosteroids (Anoshchenko et al., 2021a).

Although K_p,uu,fetal_ can be estimated at term from UV/MP values, sampling UV blood is not possible at earlier gestational ages. Therefore, to estimate drug K_p,uu,fetal_ at earlier gestational ages, the only recourse is PBPK modeling and simulation. For all the above reasons, we estimated K_p,uu,fetal_ of nelfinavir, efavirenz, and imatinib at term and earlier in gestation (nelfinavir and efavirenz only). In addition, though drugs are frequently taken by pregnant women, no UV/MP data are available for the majority of these drugs. Because obtaining such data is extremely challenging, the only recourse is to estimate K_p,uu,fetal_ for these drugs. We have previously shown that this is possible through in vitro transport studies combined with m-f-PBPK modeling and simulation and the quantitative targeted proteomics-informed relative expression factor (REF) approach (Anoshchenko et al., 2021b). However, such predictive methods need to be validated. Thus, another reason for estimating term nelfinavir, efavirenz, and imatinib K_p,uu,fetal_ values was to use them in the future to validate predictions made by our m-f-PBPK model (Anoshchenko et al., 2021b).

K_p,uu,fetal_ is determined by several factors, namely placental transport (efflux or influx), placental metabolism, and fetal clearance of the drug. Since the placenta is not endowed with the CYP450 enzymes found in adult livers, the metabolism of most drugs within this organ is negligible (Unadkat et al., 2004). The fetal liver size is small. In addition, except for CYP3A7, it also does not express many of the CYP450 enzymes found in the adult liver until about one year after birth (Thakur et al., 2021). For both of these reasons, the fetal liver plays a miniscule role in the CYP450 clearance of drugs. Therefore, for the drugs studied here, we assumed that the placental and fetal metabolism of these drugs was negligible. Consequently, as we have shown before, K_p,uu,fetal_ of these drugs will be determined solely by passive diffusion and transport across the placenta (Zhang et al., 2017).

To estimate K_p,uu,fetal_, we deliberately used the UV/MP values as our endpoint rather than just the UV unbound plasma AUC profile. This is because the latter is determined by maternal unbound plasma concentrations that are highly variable (see Fig. 5), resulting in highly variable UV plasma concentrations (total and unbound). This high variability is due to pooling UV and MP values from multiple maternal-fetal dyads. Using UV/MP values as an endpoint mitigates the variability observed when using the UV values as endpoints.

In the present study, the PK parameters of three drugs, effluxed by the placental transporters, were successfully predicted and validated after PBPK modeling and simulation of PK data in nonpregnant adults and pregnant women (Tables 2–4). Then, the K_p,uu,fetal_ of these drugs at term was estimated to be 0.41, 0.39, and 0.35 for nelfinavir, efavirenz, and imatinib, respectively. The fraction of these drugs effluxed by the placenta (f_efflux_ = 1 − K_p,uu,fetal_) was 0.59, 0.61, and 0.65, respectively, demonstrating that placental P-gp and BCRP significantly prevent their distribution into the fetal compartment. To our knowledge, this is the first time that the K_p,uu,fetal_ of a placental BCRP substrate as well as that of a dual P-gp/BCRP substrate have been estimated. Furthermore, this is the first study to construct and validate a PBPK model for the disposition of nelfinavir in nonpregnant adults and pregnant women.

Based on the above term pregnancy data, because we have quantified the abundance of placental transporters at various gestational ages (Anoshchenko et al., 2020), we were able to predict the K_p,uu,fetal_ of nelfinavir and efavirenz earlier in gestation (GW15 and GW25). The Simcyp pregnancy module does not allow predictions any earlier (<GW15), as physiologic data at these earlier gestational ages are not currently available. In addition, we could not make these predictions for imatinib, as the f_efflux_ of this drug by placental P-gp and BCRP is currently not known. However, these values can be predicted in the future from in vitro transport data and REF, as we have done before for other drugs (Kumar et al., 2021). Consistent with our expectations and previous publication (Anoshchenko et al., 2021a), due to a decrease in placental size, both CL_efflux,placenta_ and CL_PD,placenta_ decreased with gestational age, but the decrease in the latter was greater than the former. Therefore, the K_p,uu,fetal_ of both nelfinavir and efavirenz at GW15 (0.23, 0.27) and GW25 (0.34, 0.33) was lower than at term (0.41, 0.39). These data can inform the fetal efficacy and toxicity of these drugs at earlier gestational ages.

There are a few limitations to our study. First, the PBPK model of imatinib was not validated for pregnant women due to a lack of such in vivo data. Second, imatinib may be transported by human organic anion transporting polypeptide 1A2 (OATP1A2) and multidrug resistance protein 4 (MRP4) (Hu et al., 2008; Yamakawa et al., 2011). However, data on pregnancy-induced changes in OATP1A2 and MRP4 activity are not available and therefore were not included in our model based on Adiwidjaja’s model (Adiwidjaja et al., 2020). Third, for our nelfinavir PBPK model, f_m_ by each CYP450 isoform was based on CYP450 inhibition of nelfinavir metabolism in HLMs, and enzyme cross-inhibition by these inhibitors was not taken into consideration (Patilea-Vrana et al., 2019). However, none of the above limitations detracts from correctly estimating K_p,uu,fetal_, provided that the maternal plasma concentrations are predicted well. Fourth, we assumed that nelfinavir solely binds to AAG rather than albumin (I), as the association constant of nelfinavir for AAG (7.25 × 10^7^/M) is 70 times higher than that for HSA (1.11 × 10^6^/M) (Motoya et al., 2006). Fifth, the fraction unbound of the drugs in fetal plasma was the Simcyp-predicted value (Supplemental Table 2) because the corresponding experimentally measured values are not available in the literature. Any inaccuracy in our estimate of the fraction of drug bound in the maternal and fetal compartment will result in inaccuracy in our K_p,uu,fetal_ estimate. Sixth, the potential effects of HIV or cancer comorbidity on the placental drug permeability or transporters are unknown and were therefore not incorporated in the model. Again, this does not detract from our estimate of K_p,uu,fetal_, as it was based on the observed data from women who had these clinical conditions. Seventh, the Simcyp model does not allow passage of drug from the placenta directly into the amniotic fluid, which can be swallowed by the fetus. Irrespective of the route of drug passage, our K_p,uu,fetal_ values will be unaffected, as they are based on the observed UV/MP values.

In summary, we estimated the in vivo K_p,uu,fetal_ of nelfinavir, efavirenz, and imatinib through PBPK modeling and simulation. Prospectively, the K_p,uu,fetal_ of these drugs could be used to design dosing regimens of these drugs for pregnant women throughout pregnancy to maximize their efficacy and minimize their fetal toxicity. Furthermore, in the future, these K_p,uu,fetal_ could be used to validate their predictions made through in vitro studies using the proteomics-informed REF approach. Once validated, these m-f-PBPK models, in combination with in vitro studies, could be used in the future to predict fetal exposure throughout pregnancy to any drug that is actively effluxed by placental P-gp or BCRP.

## Abbreviations

AAFE: absolute average fold error
AAG: *α*1-acid glycoprotein
AUC: area under the curve of the total plasma concentration-time profile
AUC_fetal_: area under the curve of the umbilical vein total plasma concentration-time profile
AUC_m_: area under the curve of the maternal total plasma concentration-time profile
BCRP: breast cancer resistance protein
CL_efflux, placenta_: placental efflux clearance
CL_int_: intrinsic clearance
CL_int, efflux, placenta_: intrinsic placental efflux clearance
CL_int, PD, placenta_: intrinsic placental passive diffusion clearance
CL_PD_: passive diffusion clearance
CL_PD, placenta_: placental passive diffusion clearance
C-T profile: drug concentration-time profile
CYP450: cytochrome P450
f_efflux_: fraction of drug transported by placental P-gp or BCRP
f_m_: fraction of drug metabolized
GW: gestational week
HIV: human immunodeficiency virus
HLM: human liver microsome
K_p, uu, fetal_: fetal-to-maternal unbound steady-state plasma concentration ratio
m-f-PBPK model: maternal-fetal physiologically based pharmacokinetic model
MP: maternal plasma
P_app_: apparent permeability
P-gp: P-glycoprotein
PK: pharmacokinetics
REF: relative expression factor
UV: umbilical vein

## References

[B1] Adiwidjaja J Boddy AV McLachlan AJ (2020) Implementation of a physiologically based pharmacokinetic modeling approach to guide optimal dosing regimens for imatinib and potential drug interactions in paediatrics. Front Pharmacol 10:1672. 32082165 10.3389/fphar.2019.01672 PMC7002565

[B2] Ali R Ozkalemkas F Kimya Y Koksal N Ozkocaman V Gulten T Yorulmaz H Tunali A (2009) Imatinib use during pregnancy and breast feeding: a case report and review of the literature. Arch Gynecol Obstet 280:169–175. 19083009 10.1007/s00404-008-0861-7

[B3] Anoshchenko O Milad MA Unadkat JD (2021a) Estimating fetal exposure to the P-gp substrates, corticosteroids, by PBPK modeling to inform prevention of neonatal respiratory distress syndrome. CPT Pharmacometrics Syst Pharmacol 10:1057–1070. 34273255 10.1002/psp4.12674 PMC8452292

[B4] Anoshchenko O Prasad B Neradugomma NK Wang J Mao Q Unadkat JD (2020) Gestational age-dependent abundance of human placental transporters as determined by quantitative targeted proteomics. Drug Metab Dispos 48:735–741. 32591415 10.1124/dmd.120.000067 PMC7469251

[B5] Anoshchenko O Storelli F Unadkat JD (2021b) Successful prediction of human fetal exposure to P-glycoprotein substrate drugs using the proteomics-informed relative expression factor approach and PBPK modeling and simulation. Drug Metab Dispos 49:919–928. 34426410 10.1124/dmd.121.000538 PMC8626637

[B6] Atoyebi SA Rajoli RKR Adejuyigbe E Owen A Bolaji O Siccardi M Olagunju A (2019) Using mechanistic physiologically-based pharmacokinetic models to assess prenatal drug exposure: thalidomide versus efavirenz as case studies. Eur J Pharm Sci 140:105068. 31518681 10.1016/j.ejps.2019.105068 PMC6853277

[B7] Breedveld P Pluim D Cipriani G Wielinga P van Tellingen O Schinkel AH Schellens JHM (2005) The effect of Bcrp1 (Abcg2) on the in vivo pharmacokinetics and brain penetration of imatinib mesylate (Gleevec): implications for the use of breast cancer resistance protein and P-glycoprotein inhibitors to enable the brain penetration of imatinib in patients. Cancer Res 65:2577–2582. 15805252 10.1158/0008-5472.CAN-04-2416

[B8] Burger H van Tol H Boersma AWM Brok M Wiemer EAC Stoter G Nooter K (2004) Imatinib mesylate (STI571) is a substrate for the breast cancer resistance protein (BCRP)/ABCG2 drug pump. Blood 104:2940–2942. 15251980 10.1182/blood-2004-04-1398

[B9] Chapa R Li CY Basit A Thakur A Ladumor MK Sharma S Singh S Selen A Prasad B (2020) Contribution of uptake and efflux transporters to oral pharmacokinetics of furosemide. ACS Omega 5:32939–32950. 33403255 10.1021/acsomega.0c03930 PMC7774078

[B10] Chelysheva E Turkina A Polushkina E Shmakov R Zeifman A Aleshin S Shokhin I Guranda D Oksenjuk O Mordanov S, (2018) Placental transfer of tyrosine kinase inhibitors used for chronic myeloid leukemia treatment. Leuk Lymphoma 59:733–738. 28703026 10.1080/10428194.2017.1347929

[B11] Cressey TR Stek A Capparelli E Bowonwatanuwong C Prommas S Sirivatanapa P Yuthavisuthi P Neungton C Huo Y Smith E, ; IMPAACT P1026s Team (2012) Efavirenz pharmacokinetics during the third trimester of pregnancy and postpartum. J Acquir Immune Defic Syndr 59:245–252. 22083071 10.1097/QAI.0b013e31823ff052 PMC3288559

[B12] Damle B Hewlett D Jr Hsyu PH Becker M Petersen A (2006) Pharmacokinetics of nelfinavir in subjects with hepatic impairment. J Clin Pharmacol 46:1241–1249. 17050789 10.1177/0091270006292164

[B13] Dickinson L Amin J Else L Boffito M Egan D Owen A Khoo S Back D Orrell C Clarke A, (2016) Comprehensive pharmacokinetic, pharmacodynamic and pharmacogenetic evaluation of once-daily efavirenz 400 and 600 mg in treatment-naïve HIV-infected patients at 96 weeks: results of the ENCORE1 study. Clin Pharmacokinet 55:861–873. 26715213 10.1007/s40262-015-0360-5 PMC4916189

[B14] Dickmann LJ Isoherranen N (2013) Quantitative prediction of CYP2B6 induction by estradiol during pregnancy: potential explanation for increased methadone clearance during pregnancy. Drug Metab Dispos 41:270–274. 22815312 10.1124/dmd.112.047118

[B15] Dirson G Fernandez C Hindlet P Roux F German-Fattal M Gimenez F Farinotti R (2006) Efavirenz does not interact with the ABCB1 transporter at the blood-brain barrier. Pharm Res 23:1525–1532. 16779703 10.1007/s11095-006-0279-5

[B16] Dixit V Hariparsad N Li F Desai P Thummel KE Unadkat JD (2007) Cytochrome P450 enzymes and transporters induced by anti-human immunodeficiency virus protease inhibitors in human hepatocytes: implications for predicting clinical drug interactions. Drug Metab Dispos 35:1853–1859. 17639026 10.1124/dmd.107.016089

[B17] Dutreix C Peng B Mehring G Hayes M Capdeville R Pokorny R Seiberling M (2004) Pharmacokinetic interaction between ketoconazole and imatinib mesylate (Glivec) in healthy subjects. Cancer Chemother Pharmacol 54:290–294. 15138710 10.1007/s00280-004-0832-z

[B18] Fang A Valluri SR O’Sullivan MJ Maupin R Jones T Delke I Clax P (2012) Safety and pharmacokinetics of nelfinavir during the second and third trimesters of pregnancy and postpartum. HIV Clin Trials 13:46–59. 22306587 10.1310/hct1301-046

[B19] Gertz M Harrison A Houston JB Galetin A (2010) Prediction of human intestinal first-pass metabolism of 25 CYP3A substrates from in vitro clearance and permeability data. Drug Metab Dispos 38:1147–1158. 20368326 10.1124/dmd.110.032649

[B20] Gupta A Zhang Y Unadkat JD Mao Q (2004) HIV protease inhibitors are inhibitors but not substrates of the human breast cancer resistance protein (BCRP/ABCG2). J Pharmacol Exp Ther 310:334–341. 15007102 10.1124/jpet.104.065342

[B21] Haas DM Marsh DJ Dang DT Parker CB Wing DA Simhan HN Grobman WA Mercer BM Silver RM Hoffman MK, (2018) Prescription and other medication use in pregnancy. Obstet Gynecol 131:789–798. 29630018 10.1097/AOG.0000000000002579 PMC5912972

[B22] Hamada A Miyano H Watanabe H Saito H (2003) Interaction of imatinib mesilate with human P-glycoprotein. J Pharmacol Exp Ther 307:824–828. 12975485 10.1124/jpet.103.055574

[B23] Hirt D Urien S Jullien V Firtion G Chappuy H Rey E Pons G Mandelbrot L Treluyer JM (2007) Pharmacokinetic modelling of the placental transfer of nelfinavir and its M8 metabolite: a population study using 75 maternal-cord plasma samples. Br J Clin Pharmacol 64:634–644. 17892516 10.1111/j.1365-2125.2007.02885.x PMC2203265

[B24] Hu S Franke RM Filipski KK Hu C Orwick SJ de Bruijn EA Burger H Baker SD Sparreboom A (2008) Interaction of imatinib with human organic ion carriers. Clin Cancer Res 14:3141–3148. 18483382 10.1158/1078-0432.CCR-07-4913

[B25] Janneh O Chandler B Hartkoorn R Kwan WS Jenkinson C Evans S Back DJ Owen A Khoo SH (2009) Intracellular accumulation of efavirenz and nevirapine is independent of P-glycoprotein activity in cultured CD4 T cells and primary human lymphocytes. J Antimicrob Chemother 64:1002–1007. 19748977 10.1093/jac/dkp335

[B26] Kaeser B Charoin JE Gerber M Oxley P Birnboeck H Saiedabadi N Banken L (2005) Assessment of the bioequivalence of two nelfinavir tablet formulations under fed and fasted conditions in healthy subjects. Int J Clin Pharmacol Ther 43:154–162. 15792400 10.5414/cpp43154

[B27] Kapraun DF Wambaugh JF Setzer RW Judson RS (2019) Empirical models for anatomical and physiological changes in a human mother and fetus during pregnancy and gestation. PLoS One 14:e0215906. 31048866 10.1371/journal.pone.0215906 PMC6497258

[B28] Ke AB Nallani SC Zhao P Rostami-Hodjegan A Isoherranen N Unadkat JD (2013) A physiologically based pharmacokinetic model to predict disposition of CYP2D6 and CYP1A2 metabolized drugs in pregnant women. Drug Metab Dispos 41:801–813. 23355638 10.1124/dmd.112.050161 PMC3608458

[B29] Ke AB Nallani SC Zhao P Rostami-Hodjegan A Unadkat JD (2012) A PBPK model to predict disposition of CYP3A-metabolized drugs in pregnant women: verification and discerning the site of CYP3A induction. CPT Pharmacometrics Syst Pharmacol 1:e3. 23835883 10.1038/psp.2012.2 PMC3606941

[B30] Ke AB Nallani SC Zhao P Rostami-Hodjegan A Unadkat JD (2014) Expansion of a PBPK model to predict disposition in pregnant women of drugs cleared via multiple CYP enzymes, including CYP2B6, CYP2C9 and CYP2C19. Br J Clin Pharmacol 77:554–570. 23834474 10.1111/bcp.12207 PMC4371535

[B31] Kim RB Fromm MF Wandel C Leake B Wood AJJ Roden DM Wilkinson GR (1998) The drug transporter P-glycoprotein limits oral absorption and brain entry of HIV-1 protease inhibitors. J Clin Invest 101:289–294. 9435299 10.1172/JCI1269 PMC508566

[B32] Kirby BJ Collier AC Kharasch ED Whittington D Thummel KE Unadkat JD (2011) Complex drug interactions of HIV protease inhibitors 1: inactivation, induction, and inhibition of cytochrome P450 3A by ritonavir or nelfinavir. Drug Metab Dispos 39:1070–1078. 21406602 10.1124/dmd.110.037523 PMC3100903

[B33] Kreitchmann R Schalkwijk S Best B Wang J Colbers A Stek A Shapiro D Cressey T Mirochnick M Burger D (2019) Efavirenz pharmacokinetics during pregnancy and infant washout. Antivir Ther 24:95–103. 30530925 10.3851/IMP3283 PMC6642905

[B34] Kumar V Yin M Ishida K Salphati L Hop CECA Rowbottom C Xiao G Lai Y Mathias A Chu X, (2021) Prediction of transporter-mediated rosuvastatin hepatic uptake clearance and drug Interaction in humans using proteomics-informed REF approach. Drug Metab Dispos 49:159–168. 33051248 10.1124/dmd.120.000204

[B35] Kurowski M Kaeser B Sawyer A Popescu M Mrozikiewicz A (2002) Low-dose ritonavir moderately enhances nelfinavir exposure. Clin Pharmacol Ther 72:123–132. 12189359 10.1067/mcp.2002.126178

[B36] Ladumor MK Bhatt DK Gaedigk A Sharma S Thakur A Pearce RE Leeder JS Bolger MB Singh S Prasad B (2019a) Ontogeny of hepatic sulfotransferases and prediction of age-dependent fractional contribution of sulfation in acetaminophen metabolism. Drug Metab Dispos 47:818–831. 31101678 10.1124/dmd.119.086462 PMC6614793

[B37] Ladumor MK Thakur A Sharma S Rachapally A Mishra S Bobe P Rao VK Pammi P Kangne H Levi D, (2019b) A repository of protein abundance data of drug metabolizing enzymes and transporters for applications in physiologically based pharmacokinetic (PBPK) modelling and simulation. Sci Rep 9:9709. 31273226 10.1038/s41598-019-45778-9 PMC6609630

[B38] Lamorde M Wang X Neary M Bisdomini E Nakalema S Byakika-Kibwika P Mukonzo JK Khan W Owen A McClure M, (2018) Pharmacokinetics, pharmacodynamics, and pharmacogenetics of efavirenz 400 mg once daily during pregnancy and post-partum. Clin Infect Dis 67:785–790. 30124823 10.1093/cid/ciy161

[B39] Lillibridge JH Liang BH Kerr BM Webber S Quart B Shetty BV Lee CA (1998) Characterization of the selectivity and mechanism of human cytochrome P450 inhibition by the human immunodeficiency virus-protease inhibitor nelfinavir mesylate. Drug Metab Dispos 26:609–616. 9660842

[B40] Longer M Shetty B Zamansky I Tyle P (1995) Preformulation studies of a novel HIV protease inhibitor, AG1343. J Pharm Sci 84:1090–1093. 8537887 10.1002/jps.2600840911

[B41] Mahar Doan KM Humphreys JE Webster LO Wring SA Shampine LJ Serabjit-Singh CJ Adkison KK Polli JW (2002) Passive permeability and P-glycoprotein-mediated efflux differentiate central nervous system (CNS) and non-CNS marketed drugs. J Pharmacol Exp Ther 303:1029–1037. 12438524 10.1124/jpet.102.039255

[B42] McGowan JP Shah SS (2000) Prevention of perinatal HIV transmission during pregnancy. J Antimicrob Chemother 46:657–668. 11062184 10.1093/jac/46.5.657

[B43] Mitchell AA Gilboa SM Werler MM Kelley KE Louik C Hernández-Díaz S; National Birth Defects Prevention Study (2011) Medication use during pregnancy, with particular focus on prescription drugs: 1976-2008. Am J Obstet Gynecol 205:51.e1–51.e8. 21514558 10.1016/j.ajog.2011.02.029 PMC3793635

[B44] Motoya T Thevanayagam LN Blaschke TF Au S Stone JA Jayewardene AL Chi J Aweeka FT (2006) Characterization of nelfinavir binding to plasma proteins and the lack of drug displacement interactions. HIV Med 7:122–128. 16420257 10.1111/j.1468-1293.2006.00356.x

[B45] Oostendorp RL Buckle T Beijnen JH van Tellingen O Schellens JHM (2009) The effect of P-gp (Mdr1a/1b), BCRP (Bcrp1) and P-gp/BCRP inhibitors on the in vivo absorption, distribution, metabolism and excretion of imatinib. Invest New Drugs 27:31–40. 18449471 10.1007/s10637-008-9138-z

[B46] Ostrowicz A Mikołajczak PL Wierzbicka M Boguradzki P (2014) Bioequivalence study of 400 and 100 mg imatinib film-coated tablets in healthy volunteers. Acta Pol Pharm 71:843–854. 25362813

[B47] Patilea-Vrana GI Anoshchenko O Unadkat JD (2019) Hepatic enzymes relevant to the disposition of (-)-Δ^9^-tetrahydrocannabinol (THC) and its psychoactive metabolite, 11-OH-THC. Drug Metab Dispos 47:249–256. 30567877 10.1124/dmd.118.085548 PMC6374540

[B48] Peng B Dutreix C Mehring G Hayes MJ Ben-Am M Seiberling M Pokorny R Capdeville R Lloyd P (2004) Absolute bioavailability of imatinib (Glivec) orally versus intravenous infusion. J Clin Pharmacol 44:158–162. 14747424 10.1177/0091270003262101

[B49] Peroni RN Di Gennaro SS Hocht C Chiappetta DA Rubio MC Sosnik A Bramuglia GF (2011) Efavirenz is a substrate and in turn modulates the expression of the efflux transporter ABCG2/BCRP in the gastrointestinal tract of the rat. Biochem Pharmacol 82:1227–1233. 21803024 10.1016/j.bcp.2011.07.081

[B50] Perry CM Frampton JE McCormack PL Siddiqui MAA Cvetković RS (2005) Nelfinavir: a review of its use in the management of HIV infection. Drugs 65:2209–2244. 16225378 10.2165/00003495-200565150-00015

[B51] Poulin P Theil FP (2009) Development of a novel method for predicting human volume of distribution at steady-state of basic drugs and comparative assessment with existing methods. J Pharm Sci 98:4941–4961. 19455625 10.1002/jps.21759

[B52] Read JS Best BM Stek AM Hu C Capparelli EV Holland DT Burchett SK Smith ME Sheeran EC Shearer WT, (2008) Pharmacokinetics of new 625 mg nelfinavir formulation during pregnancy and postpartum. HIV Med 9:875–882. 18795962 10.1111/j.1468-1293.2008.00640.x PMC2732353

[B53] Salama NN Kelly EJ Bui T Ho RJY (2005) The impact of pharmacologic and genetic knockout of P-glycoprotein on nelfinavir levels in the brain and other tissues in mice. J Pharm Sci 94:1216–1225. 15858856 10.1002/jps.20344

[B54] Sarapa N Hsyu PH Lappin G Garner RC (2005) The application of accelerator mass spectrometry to absolute bioavailability studies in humans: simultaneous administration of an intravenous microdose of 14C-nelfinavir mesylate solution and oral nelfinavir to healthy volunteers. J Clin Pharmacol 45:1198–1205. 16172185 10.1177/0091270005280051

[B55] Shono Y Jantratid E Dressman JB (2011) Precipitation in the small intestine may play a more important role in the in vivo performance of poorly soluble weak bases in the fasted state: case example nelfinavir. Eur J Pharm Biopharm 79:349–356. 21527341 10.1016/j.ejpb.2011.04.005

[B56] Siccardi M Almond L Schipani A Csajka C Marzolini C Wyen C Brockmeyer NH Boffito M Owen A Back D (2012) Pharmacokinetic and pharmacodynamic analysis of efavirenz dose reduction using an in vitro-in vivo extrapolation model. Clin Pharmacol Ther 92:494–502. 22805423 10.1038/clpt.2012.61

[B57] Takano R Sugano K Higashida A Hayashi Y Machida M Aso Y Yamashita S (2006) Oral absorption of poorly water-soluble drugs: computer simulation of fraction absorbed in humans from a miniscale dissolution test. Pharm Res 23:1144–1156. 16715363 10.1007/s11095-006-0162-4

[B58] Thakur A Parvez MM Leeder JS Prasad B (2021) Ontogeny of drug-metabolizing enzymes. Methods Mol Biol 2342:551–593. 34272706 10.1007/978-1-0716-1554-6_18

[B59] Tolle-Sander S Rautio J Wring S Polli JW Polli JE (2003) Midazolam exhibits characteristics of a highly permeable P-glycoprotein substrate. Pharm Res 20:757–764. 12751631 10.1023/a:1023433502647

[B60] Unadkat JD Dahlin A Vijay S (2004) Placental drug transporters. Curr Drug Metab 5:125–131. 14965255 10.2174/1389200043489171

[B61] van Heeswijk RPG Khaliq Y Gallicano KD Bourbeau M Seguin I Phillips EJ Cameron DW (2004) The pharmacokinetics of nelfinavir and M8 during pregnancy and post partum. Clin Pharmacol Ther 76:588–597. 15592330 10.1016/j.clpt.2004.08.011

[B62] Villani P Regazzi MB Castelli F Viale P Torti C Seminari E Maserati R (1999) Pharmacokinetics of efavirenz (EFV) alone and in combination therapy with nelfinavir (NFV) in HIV-1 infected patients. Br J Clin Pharmacol 48:712–715. 10594473 10.1046/j.1365-2125.1999.00071.x PMC2014352

[B63] Vrouenraets SME Wit FWNM van Tongeren J Lange JMA (2007) Efavirenz: a review. Expert Opin Pharmacother 8:851–871. 17425480 10.1517/14656566.8.6.851

[B64] Yamakawa Y Hamada A Shuto T Yuki M Uchida T Kai H Kawaguchi T Saito H (2011) Pharmacokinetic impact of SLCO1A2 polymorphisms on imatinib disposition in patients with chronic myeloid leukemia. Clin Pharmacol Ther 90:157–163. 21633340 10.1038/clpt.2011.102

[B65] Yamashita S Furubayashi T Kataoka M Sakane T Sezaki H Tokuda H (2000) Optimized conditions for prediction of intestinal drug permeability using Caco-2 cells. Eur J Pharm Sci 10:195–204. 10767597 10.1016/s0928-0987(00)00076-2

[B66] Zhang KE Wu E Patick AK Kerr B Zorbas M Lankford A Kobayashi T Maeda Y Shetty B Webber S (2001) Circulating metabolites of the human immunodeficiency virus protease inhibitor nelfinavir in humans: structural identification, levels in plasma, and antiviral activities. Antimicrob Agents Chemother 45:1086–1093. 11257019 10.1128/AAC.45.4.1086-1093.2001 PMC90428

[B67] Zhang Z Farooq M Prasad B Grepper S Unadkat JD (2015) Prediction of gestational age-dependent induction of in vivo hepatic CYP3A activity based on HepaRG cells and human hepatocytes. Drug Metab Dispos 43:836–842. 25802327 10.1124/dmd.114.062984 PMC4429679

[B68] Zhang Z Imperial MZ Patilea-Vrana GI Wedagedera J Gaohua L Unadkat JD (2017) Development of a novel maternal-fetal physiologically based pharmacokinetic model I: insights into factors that determine fetal drug exposure through simulations and sensitivity analyses. Drug Metab Dispos 45:920–938. 28588050 10.1124/dmd.117.075192 PMC5506457

[B69] Zhang Z Unadkat JD (2017) Development of a novel maternal-fetal physiologically based pharmacokinetic model II: verification of the model for passive placental permeability drugs. Drug Metab Dispos 45:939–946. 28049636 10.1124/dmd.116.073957 PMC5506455

[B70] Zhou L Schmidt K Nelson FR Zelesky V Troutman MD Feng B (2009) The effect of breast cancer resistance protein and P-glycoprotein on the brain penetration of flavopiridol, imatinib mesylate (Gleevec), prazosin, and 2-methoxy-3-(4-(2-(5-methyl-2-phenyloxazol-4-yl)ethoxy)phenyl)propanoic acid (PF-407288) in mice. Drug Metab Dispos 37:946–955. 19225039 10.1124/dmd.108.024489

